# Peak myocardial work index: a novel nonproprietary approach for the assessment of global constructive myocardial work by strain echocardiography

**DOI:** 10.1007/s10554-025-03522-7

**Published:** 2025-09-26

**Authors:** Peng Chen, Christina Kiriakou, Matthias Aurich, Sebastian Greiner, Gabriele Maliandi, Matthias Müller-Hennessen, Evangelos Giannitsis, Benjamin Meder, Norbert Frey, Sven Pleger, Derliz Mereles

**Affiliations:** Department of Internal Medicine III, Cardiology, Angiology and Pneumology, University of Heidelberg, Heidelberg, Germany

**Keywords:** Speckle-tracking echocardiography, Global longitudinal strain, Myocardial work indices, Peak myocardial work index, Adverse outcomes, Dilated cardiomyopathy

## Abstract

Myocardial work indices (MW) have been validated with respect to their efficiency for predicting cardiac events in patients with heart failure. However, the measurement of MW requires specific vendor software that may not be ubiquitous accessible. We aimed to explore the feasibility of using a nonproprietary method, peak myocardial work index (PMW) = systolic blood pressure * global longitudinal strain, as a potential substitute to global constructive work (GCW) for the assessment of left ventricular function. A retrospective analysis of 116 patients with dilated cardiomyopathy (DCM) and an equal number of age- and sex-matched healthy controls examined from June 2009 to July 2014 was conducted. Compared to healthy controls, the PMW index and GCW were significantly lower in DCM patients: 1371 ± 541 vs. 2520 ± 361 mm Hg%, 1318 ± 502 vs. 2322 ± 333 mm Hg%, respectively (*p* < 0.001 for each). Additionally, PMW showed an excellent correlation with GCW (*r* = 0.99, *p* < 0.001). During a mean follow-up time of 5.1 years, 34 patients (29.3%) reached the composite endpoints: 5 patients received cardiac transplantation, 17 patients were hospitalized due to heart failure, 9 patients received appropriate ICD therapy and 3 patients died. PMW per 50 mm Hg% increase (HR = 0.92, 95%CI 0.89–0.96, *p* < 0.001) and GCW per 50 mm Hg% increase (HR = 0.91, 95%CI 0.88–0.95, *p* < 0.001) performed comparably in predicting adverse outcomes in DCM patients in the univariate Cox regression analyses. PMW and GCW were the independent prognostic factors after adjusting for significant parameters of the univariate analysis. Patients with PMW < 1,286 mm Hg% (HR = 3.71, 95%CI 1.18–11.63, *p* = 0.025) and GCW < 1,238 mm Hg% (HR = 4.8, 95%CI 1.57–14.68, *p* = 0.006) had higher risks of MACE. PMW index might serve as an alternative echocardiographic method for evaluating left ventricular systolic function, providing similar diagnostic and prognostic capacity comparable to GCW.

## Introduction

Heart failure (HF) is a complex clinical syndrome characterized by the inability of a structurally and/or functionally abnormal heart to pump sufficient blood to meet the body’s metabolic needs. The etiology of HF is various, encompassing a range of cardiovascular disease: coronary artery disease, hypertension, valvular heart disease and cardiomyopathy [1]. HF is also a growing public health concern worldwide, with its prevalence increasing with age in adult population, estimated to be around 1–3% [2–4]. Acute heart failure (AHF) can present as either a new-onset condition or more commonly as acute decompensation of chronic HF, which is often associated with hospitalization, morbidity, and mortality [5–8]. These facts underscore the importance of early detection and accurate assessment of left ventricular (LV) function.

The assessment of LV systolic function has been conventionally conducted by means of surrogate parameters. LV ejection fraction (LV-EF) by echocardiography has been widely used in assessment of LV function because of its simplicity and strong correlation with clinical outcomes in patients with HF [9]. However, the volumetric assessment has wide interobserver variability and can be inaccurately measured in certain conditions, such as dilated or hypertrophic cardiomyopathy, even if up-to-date real-time 3D volumetry is applied [10–12]. In recent decades, global longitudinal strain (GLS) derived from speckle tracking echocardiography (STE) has emerged as a more sensitive method for assessing LV function. GLS shows lower interobserver and intraobserver variability in evaluating regional and global longitudinal systolic LV function and is particularly useful in detecting LV contractile dysfunction, even when EF is preserved, making it valuable for the subtle detection of subclinical myocardial diseases [13]. Despite its advantages, GLS remains load-dependent, which can limit its utility in some clinical settings.

In recent years, myocardial work (MW) derived from left ventricular pressure-strain loop (LV-PSL), which accounts for both myocardial deformation and afterload, has demonstrated its efficiency and incremental value over LV-EF and GLS in predicting adverse outcomes in patients with HF and/or reduced EF [14–16]. MW integrates GLS with blood pressure by cuff manometer while considering valvular events, with systolic blood pressure (SBP) serving as an estimate of peak LV pressure. This process generates a 17-segment bull’s eye plot and LV-PSL curves with global and segmental myocardial work, including global work index (GWI), global constructive work (GCW), global wasted work (GWW), and global work efficiency (GWE) [17, 18]. However, measuring MW requires vendor-specific software, limiting its widespread application in clinical practice. Given these constraints, our study aims to evaluate the feasibility of using a simplified method, peak myocardial work index (PMW) = SBP * GLS (absolute value), as an alternative to MW for assessing LV performance.

## Methods

### Study population

169 Patients with an age of ≥ 18 years and systolic heart failure who underwent heart catheterization were retrospectively recruited from June 2009 to July 2014. Clinical and laboratory information was retrieved. If significant coronary artery disease could be ruled out by left heart catheterization and moderate to severe primary valvular left heart disease was excluded, patients were defined as having non-ischemic dilated cardiomyopathy (DCM) or non-dilated left ventricular cardiomyopathy (NDLVC). All patients with an LV ejection fraction under 45% were included. Patients with LV hypertrophy due to hypertensive heart disease and hypertrophic cardiomyopathy as well as patients with poor image quality hindering deformation analyses were excluded (*n* = 53). Finally, 116 patients with DCM were included. The follow-up data was obtained according to digitized medical records. A same number of age- and sex-matched healthy controls was obtained from healthy adults. MACE was defined as: all-cause mortality, cardiac transplantation, appropriate implantable cardioverter-defibrillator (ICD) therapy, either a shock or antitachycardia pacing, and heart failure hospitalization. The study was conducted in accordance with the Declaration of Helsinki after approval by the ethics committee of Heidelberg University, and informed consent was obtained from all participants.

### Echocardiography

A comprehensive echocardiographic examination was performed using a commercially available ultrasound machine (Vivid E9, GE Healthcare Vingmed, Trondheim, Norway) equipped with a phased array probe (M5S-D) ranging from 1.5 to 4.6 MHz, according to the guidelines of the American Society of Echocardiography [19]. Three consecutive cardiac cycles were recorded per 2D image at a sampling rate of 55 to 60 frames/sec. GLS by STE and MW index were performed using offline commercial software (EchoPAC workstation BT13, GE Healthcare, Trondheim, Norway). GLS was acquired and averaged from the apical 4-, 2-, and 3-chamber views. Endocardial borders were automatically tracked by the software to build the region of interest (ROI) which was adjusted manually if necessary. All LV segments must have been clearly visible throughout the cardiac cycle for optimal regional assessment. Foreshortening of apical views was avoided since it leads to overestimation of GLS and MW index. Intra- and interobserver variability were comparable to our former publication [15].

The MW indices were assessed by incorporating blood pressures and GLS, combining the valvular event times from mitral valve closure to mitral valve opening, and then generating a pressure-strain loop (PSL) [17]. Detailed formulas are not provided by the manufacturing company. The global MW, categorized as global work index (GWI), is calculated as an average of segmental values, the global constructive work (GCW) is the work performed by a segment during shortening in systole, the global wasted work (GWW) is calculated as work performed by a segment during lengthening in systole and during shortening in isovolumic relaxation and represents the energy not contributing to blood ejection in the circulation, the global work efficiency (GWE) is calculated as GCW divided by the sum of GCW and GWW.

### Peak myocardial work index (PMW)

**Fig. 1:**
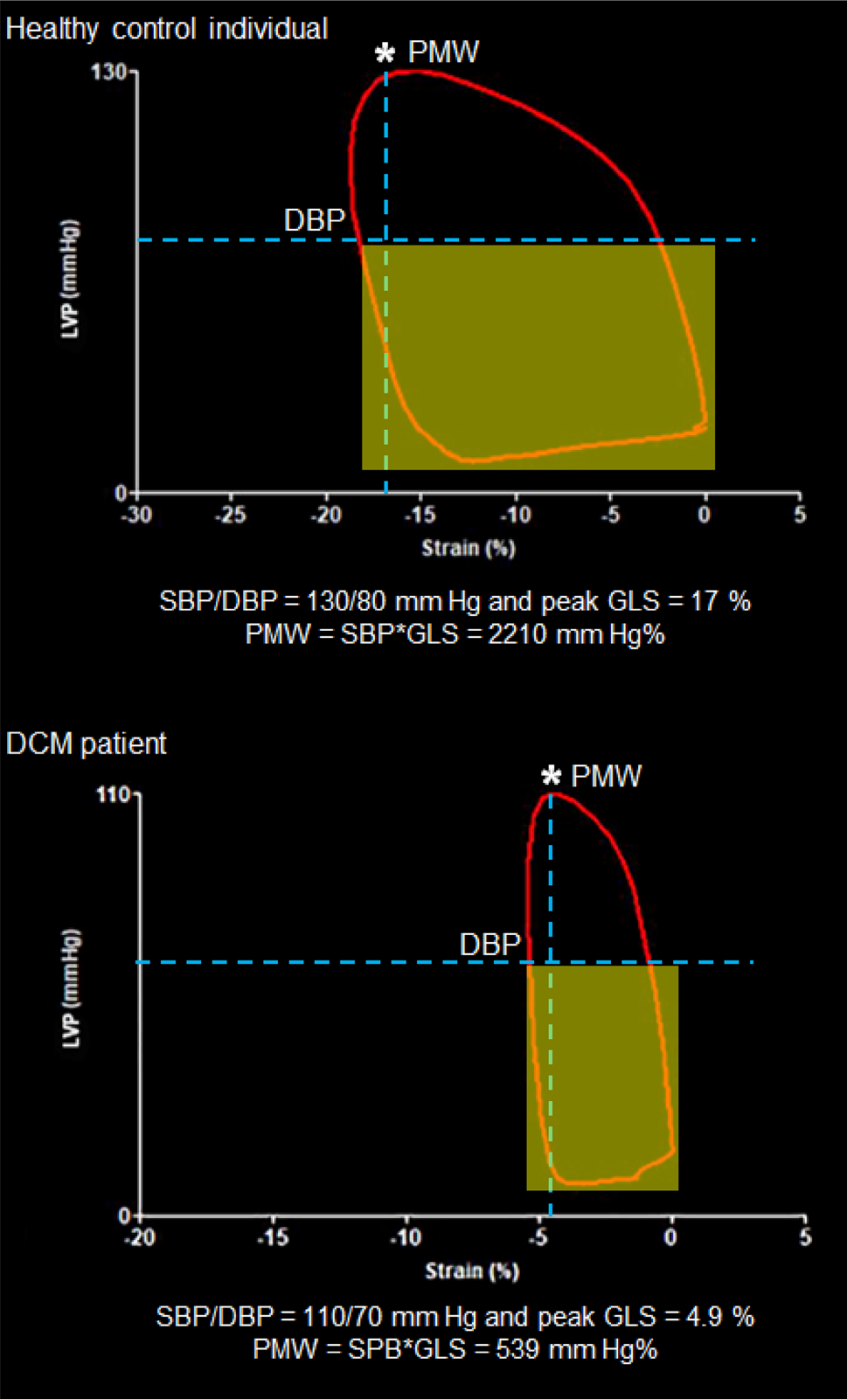
LV-PSL of a healthy control individual and a DCM patient. The vertical dash line shows PMW at the intersection of peak GLS and SBP (*). The horizontal dash line shows the DBP. The yellow marked area shows absence of LV pressure information below the level of DBP. LV-PSL: left ventricular pressure-strain loop, DCM: non-ischemic dilated cardiomyopathy, PMW: peak myocardial work index, GLS: global longitudinal strain, SBP: systolic blood pressure, DBP: diastolic blood pressure

This parameter for assessing myocardial performance was derived using a simplified formula: PMW = SBP * GLS (absolute values). Where SBP stands for peak systolic blood pressure and GLS represents the absolute values of global longitudinal strain. The peak SBP occurs approximately in mid-systole and closely approximates systolic blood pressure or central aortic pressure in the absence of significant gradient across the left ventricular outflow tract or aortic valve. Since this was a retrospective analysis, simultaneous SBP measurement were not available, however, we took caution to consider only SBP assessed in the same visit. In contrast to SBP, peak GLS occurs towards the end of systole, just before the aortic valve closes, here is when this measurement is made. These events do not occur simultaneously, however, in the LV-PSL they seem to take place at the same time GCW is measured (Fig. 1).

### Statistical analysis

All statistical analyses were performed using SPSS 26.0 for Windows (SPSS Inc., Chicago, USA). Distribution of continuous variables was assessed using the Kolmogorov-Smirnov test. Continuous variables were expressed as means ± standard deviation or medians (interquartile range) as appropriate, and analyzed using Student’s t-test is values were normally distributed, otherwise using the Mann-Whitney U-test for non-normality. Categorical variables were expressed as frequencies and percentages and analyzed using the Chi-square test or the Fisher’s exact test as appropriate. *P* < 0.05 was considered to be statistically significant. Correlations between continuous variables were assessed using Pearson’s correlation coefficient. Bland-Altman analysis was conducted to assess the agreement of PMW with GCW and GWI. Receiver operating characteristic (ROC) curve analysis was used to differentiate the DCM patients from healthy controls, as well as the patients with major adverse cardiovascular events (MACE) from those without MACE. The cut-off values for dichotomous analyses were derived by the maximum of Youden index. Univariate Cox regression analysis was performed to identify the determinants of adverse outcomes. For variables with *p* < 0.05 in univariate Cox regression were recruited to multivariate Cox regression and several multivariate Cox regression models were conducted according to clinical relevance to determine the independent factor for predicting adverse outcomes. Schoenfeld residuals were utilized to verify the proportional hazards assumption while the variance inflation factor was used to assess multicollinearity in the Cox regression analysis. Furthermore, the corresponding cut-off values were derived by the maximum of the Youden index. Subsequently, Kaplan-Meier survival curves were conducted to display the occurrence of clinical events over time.

## Results

### Characteristics of study population

A total of 116 patients with non-ischemic DCM and an equal number of sex- and age-matched healthy controls were retrospectively included in this study, conducted between June 2009 and July 2014. The mean follow-up time for DCM patients was 5.1 years. During this period, 34 patients (29.3%) met the composite endpoints: 5 patients received cardiac transplantation, 17 patients were hospitalized due to heart failure, 9 patients received appropriate ICD therapy and 3 patients died. No patients had severe valvular disease. All patients were treated according to heart failure guidelines prior to 2015, thus, without sacubitril/valsartan and without sodium-glucose co-transporter 2 inhibitors.

Clinical, laboratory, and echocardiographic parameters for the two groups are summarized in Table 1. The study predominantly included males, *n* = 87 (75%), with a mean age of 55 years.

**Table 1:** Characteristics of healthy controls and DCM patients

Parameters	Healthy controls	DCM patients	*p*
n	116	116	
**Baseline characteristics**			
Age, years	55.4 ± 13.7	55.1 ± 14.0	0.85
Male, n (%)	87 (75)	87 (75)	1
BMI, kg/m^2^	24.1 ± 2.4	25.8 ± 3.8	< 0.001
SBP, mmHg	130.7 ± 14.6	119.4 ± 17.3	< 0.001
DBP, mmHg	82.2 ± 8.7	74.7 ± 11.0	< 0.001
NYHA class I/II/III/IV, n		50/45/19/2	
**Clinical chemistry**			
NT-proBNP, pg/mL	56 (33, 98)	525 (154, 1438)	< 0.001
hs-TnT, ng/L	5 (3, 7)	11 (6, 24)	0.041
**Echocardiography**			
LV-EDD, mm	46.0 ± 3.9	57.1 ± 7.8	< 0.001
LV-ESD, mm	31.0 ± 3.6	47.8 ± 9.9	< 0.001
LV-EDV, mL	98.4 ± 19.7	161.5 ± 54.3	< 0.001
LV-ESV, mL	37.5 ± 8.3	110.7 ± 48.6	< 0.001
LV-EF, %	61.7 ± 5.6	33.0 ± 10.3	< 0.001
IVS, mm	8.9 ± 1.8	10.6 ± 2.2	< 0.001
PW, mm	8.8 ± 1.5	10.3 ± 2.0	< 0.001
LV mass index, g/m^2^	71.7 ± 17.2	124.2 ± 35.1	< 0.001
MAPSE, cm	1.5 ± 0.2	1.2 ± 0.3	< 0.001
LV-GLS, -%	19.3 ± 1.7	11.5 ± 4.4	< 0.001
GWI, mm Hg%	1,938.3 ± 282.0	1,032.0 ± 442.1	< 0.001
GCW, mm Hg%	2,321.9 ± 332.5	1,318.4 ± 502.1	< 0.001
PMW, mm Hg%	2,520.2 ± 360.5	1,370.5 ± 541.0	< 0.001
GWW, mm Hg%	96 (70, 145)	151 (105, 219)	< 0.001
GWE, %	95 (94, 96)	88 (80, 92)	< 0.001
E/e’	6.3 ± 2.0	9.0 ± 5.1	< 0.001
LAVI, mL/m^2^	25.1 ± 6.7	32.3 ± 16.1	< 0.001
RV-EDD, mm	33.4 ± 5.8	36.1 ± 7.6	0.002
RV-FAC, %	48.2 ± 9.6	33.3 ± 13.2	< 0.001
RV-FWS, -%	21.8 ± 3.1	12.9 ± 5.4	< 0.001
TAPSE, cm	2.4 ± 0.4	1.9 ± 0.5	< 0.001

BMI, body mass index; SBP, systolic blood pressure; DBP, diastolic blood pressure; NYHA, New York Heart Association classification; NT-proBNP, N-terminal prohormone of brain natriuretic peptide; hs-TnT, high-sensitive cardiac troponin T; LV, left ventricle; EDD, end-diastolic diameter; ESD, end-systolic diameter; EDV, end-diastolic volume; ESV, end-systolic volume; EF, ejection fraction, modified Simpson’s rule biplane; IVS, interventricular septum; PW, LV posterior wall; MAPSE, mitral annular plane systolic excursion; GLS, global longitudinal strain; GWI, global work index; GCW, global constructive work; PMW: peak myocardial work index; GWW, global wasted work; GWE, global work efficiency; E/e’, ratio of passive mitral inflow velocity (E) to tissue Doppler mitral annular velocity (e’); LAVI, left atrial volume index; RV, right ventricle; RV-EDD, basal RV end-diastolic diameter; RV-FAC, RV fractional area change; RV-FWS, RV free-wall longitudinal strain; TAPSE, tricuspid annular systolic plane excursion; RA, right atrium; RA-ESA, RA end-systolic area

### Correlations between PMW index and echocardiographic parameters

PMW demonstrated an excellent correlation with GCW (*r* = 0.988, *p* < 0.001) (Fig. 1A). The Bland-Altman analysis revealed a mean bias of 0.06, with 95% limits of agreement ranging from − 0.11 to 0.22 (Fig. 1B). Similarly, PMW showed a strong correlation with GWI (*r* = 0.980, *p* < 0.001) (Fig. 1C). The Bland-Altman analysis for GWI revealed a mean bias of 0.28, with 95% limits of agreement ranging from 0.10 to 0.46 (Fig. 1D). PMW showed a good correlation with LV GLS and LV-EF (*r* = 0.938, *p* < 0.001 and *r* = 0.852, *p* < 0.001, respectively) and a moderate correlation with MAPSE (*r* = 0.656, *p* < 0.001).

### Diagnostic and prognostic performance of PMW index

PMW was significantly lower in DCM patients compared to healthy controls (1370.5 ± 541.0 vs. 2520.2 ± 360.5 mm Hg%, *p* < 0.001). Similarly, compared to healthy controls, GCW, GWI, LV GLS, and MAPSE had lower values in DCM patients. Furthermore, among the DCM cohort, PMW, GCW, GWI, LV GLS, and MAPSE were notably lower in patients who experienced MACE compared to those in the event-free group (Table 2).

**Table 2:** Characteristics of patients stratified according to occurrence of MACE

Parameters	No MACE	MACE	*p*
n	82	34	
**Baseline characteristics**			
Age, years	55.1 ± 14.3	55.0 ± 13.4	0.97
Male, n (%)	67 (82)	20 (59)	0.010
BMI, kg/m^2^	26.1 ± 3.7	25.3 ± 4.0	0.35
SBP, mmHg	121.4 ± 16.5	114.4 ± 18.4	0.044
DBP, mmHg	75.8 ± 11.2	72.1 ± 10.4	0.10
NYHA class ≥ II, n (%)	40 (49)	26 (77)	0.006
**Clinical chemistry**			
NT-proBNP, pg/mL	376 (114, 913)	1206 (502, 2516)	< 0.001
hs-TnT, ng/L	10 (6, 19)	12 (7, 46)	0.16
**Echocardiography**			
LV-EDD, mm	56.0 ± 7.2	59.8 ± 8.6	0.016
LV-ESD, mm	45.8 ± 9.2	52.7 ± 9.8	< 0.001
LV-EDV, mL	152.0 ± 49.8	184.2 ± 58.5	0.003
LV-ESV, mL	99.6 ± 41.3	137.5 ± 54.8	0.001
LV-EF, %	35.5 ± 9.8	26.9 ± 8.8	< 0.001
IVS, mm	10.9 ± 2.2	10.1 ± 2.2	0.07
PW, mm	10.4 ± 1.8	10.1 ± 2.4	0.44
LV mass index, g/m^2^	120.6 ± 31.7	132.9 ± 41.4	0.09
MAPSE, cm	1.3 ± 0.3	1.1 ± 0.3	0.006
LV-GLS, -%	12.5 ± 4.2	8.9 ± 3.6	< 0.001
GWI, mm Hg%	1,144.8 ± 403.1	759.9 ± 417.6	< 0.001
GCW, mm Hg%	1,448.3 ± 464.5	1,004.9 ± 453.4	< 0.001
PMW, mm Hg%	1,508.8 ± 502.5	1,036.9 ± 487.8	< 0.001
GWW, mm Hg%	150 (109, 214)	154 (91, 271)	0.89
GWE, %	89 (84, 92)	84 (73, 90)	0.002
E/e’	7 (4, 10)	10 (7, 14)	0.001
LAVI, mL/m^2^	25 (21, 37)	32 (28, 42)	0.002
RV-EDD, mm	36.0 ± 6.7	36.4 ± 9.7	0.80
RV-FAC, %	34.2 ± 13.3	31.0 ± 12.8	0.23
RV-FWS, -%	13.8 ± 5.3	10.6 ± 4.8	0.003
TAPSE, cm	1.9 ± 0.5	1.7 ± 0.5	0.025

MACE: major adverse cardiovascular events. Table footnotes are otherwise identical to Table 1

**Fig. 2:**
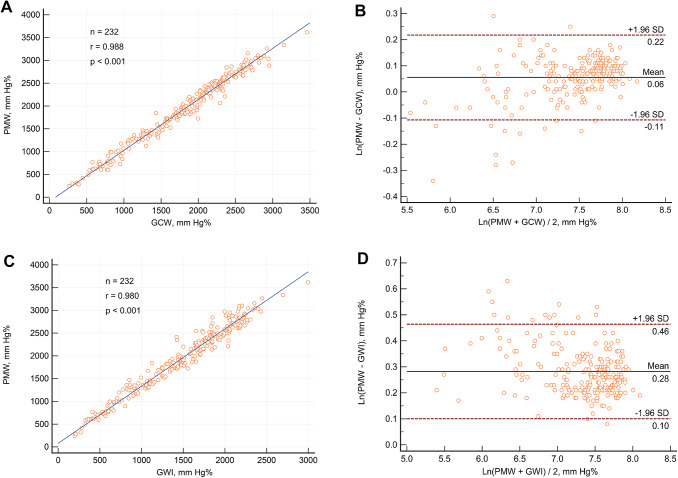
Linear regression analysis (panel A) and Bland-Altman plot (panel B) between PMW and GCW. Linear regression analysis (panel C) and Bland-Altman plot (panel D) between PMW and GWI. PWM: peak myocardial work index, GCW: global constructive work, GWI: global work index

The ability to differentiate between DCM patients and healthy controls was assessed using ROC curves, as shown in Fig. 2. PMW showed high diagnostic accuracy with AUC of 0.97, followed by GCW, GWI, absolute GLS, and MAPSE, with AUC of 0.96, 0.96, 0.95, and 0.79, respectively.

**Fig. 3:**
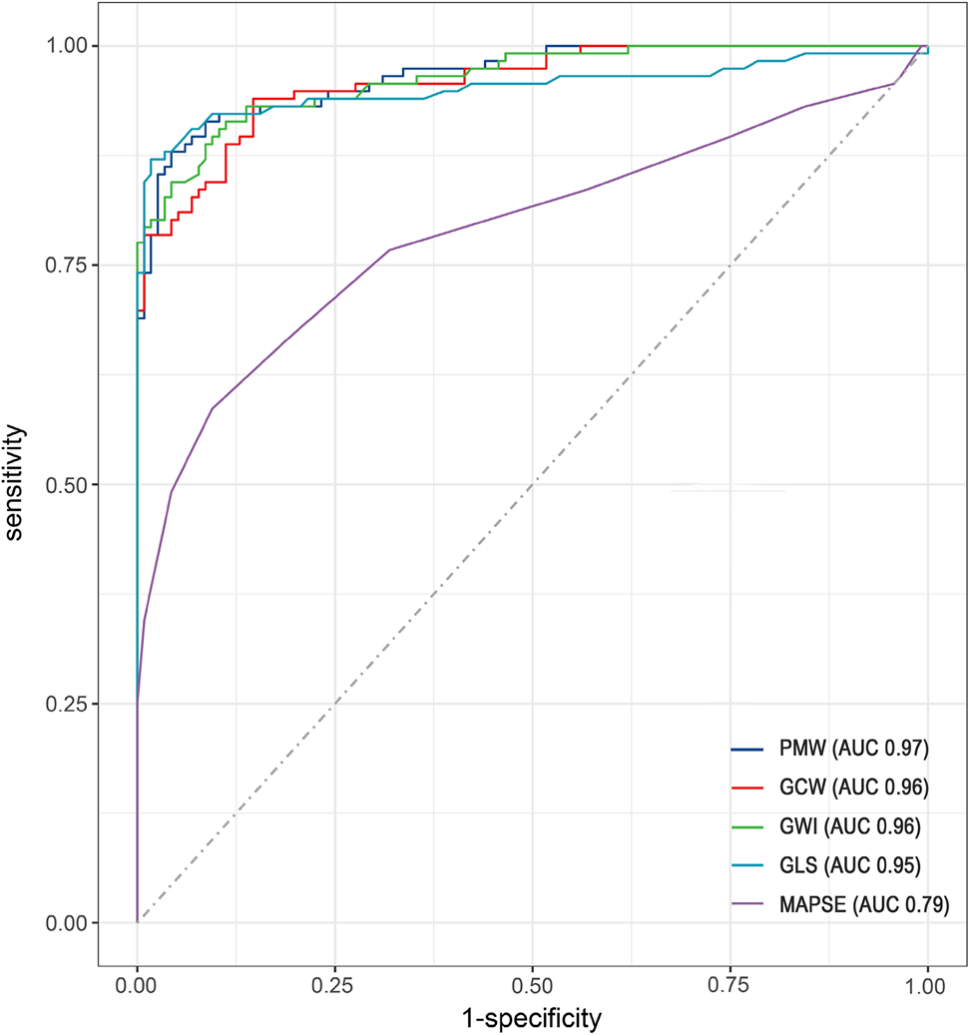
Receiver operating characteristics curve (ROC) of associations between LV functional parameters between DCM patient and healthy controls. PMW: peak myocardial work index, GCW: global constructive work, GWI: global work index, GLS: global longitudinal LV strain, MAPSE: mitral annular plane systolic excursion

The cut-off values for distinguishing patients with MACE from those without MACE were obtained using ROC curve analysis: PMW (1,286 mm Hg%), GCW (1,238 mm Hg%), GWI (788 mm Hg%), and LV GLS (−10.8%). Subsequently, Kaplan-Meier survival analyses were conducted based on these cut-off values. Patients with PMW < 1,286 mm Hg% had a higher risk of clinical events compared to those with PMW > 1,286 mm Hg% (*p* < 0.001) (Fig. 3A). Similarly, patients with GCW < 1,238 mm Hg% had a higher risk of MACE (*p* < 0.001) (Fig. 3B). Additionally, patients with lower GWI (Fig. 3C) and GLS values (Fig. 3D) also had a higher risk of events compared to those with higher values.

The results of the univariate Cox regression analysis are shown in Table 3. New York Heart Association (NYHA) functional class (≥ II), N-terminal pro–brain natriuretic peptide (≥ 681 pg/mL), and high-sensitivity troponin T (≥ 34 ng/L) were associated with clinical events. The echocardiographic parameters, PMW, GCW, GWI, LV GLS and MAPSE were predictors of adverse outcome.

**Table 3:** Univariate Cox regression analysis

Parameters	HR	95% CI	*p*
**Baseline characteristics**			
Age, years	0.998	0.974–1.023	0.88
Male, %	0.442	0.223–0.875	0.019
BMI, kg/m^2^	0.967	0.877–1.066	0.50
SBP, mm Hg	0.978	0.958–0.998	0.028
DBP, mm Hg	0.971	0.939–1.004	0.09
NYHA class, ≥II	2.728	1.234–6.029	0.013
**Clinical chemistry**			
NT-proBNP > 681, pg/mL	3.550	1.691–7.451	0.001
hs-TnT > 34, ng/L	2.457	1.192–5.066	0.015
**Echocardiography**			
LV-EDD, mm	1.060	1.016–1.105	0.007
LV-ESD, mm	1.062	1.028–1.097	< 0.001
LV-EDV, mL	1.011	1.005–1.017	0.001
LV-ESV, mL	1.015	1.008–1.021	< 0.001
LV-EF, %	0.927	0.895–0.959	< 0.001
IVS, mm	0.853	0.721–1.010	0.07
PW, mm	0.934	0.782–1.116	0.45
LV mass index, g/m^2^	1.009	1.000-1.018	0.06
MAPSE, cm	0.169	0.053–0.539	0.003
LV-GLS, -%	0.829	0.761–0.902	< 0.001
GWI, per 50 mm Hg% increase	0.901	0.862–0.942	< 0.001
GCW, per 50 mm Hg% increase	0.913	0.879–0.948	< 0.001
PMW, per 50 mm Hg% increase	0.923	0.892–0.955	< 0.001
GWW, mm Hg%	1.002	0.999–1.005	0.19
GWE, %	0.932	0.904–0.962	< 0.001
E/e’	1.085	1.034–1.139	0.001
LAVI, mL/m^2^	1.018	1.001–1.036	0.040
RV-EDD, mm	1.008	0.960–1.059	0.74
RV-FAC, %	0.202	0.014–2.836	0.24
RV-FWS, -%	0.888	0.827–0.953	0.001
TAPSE, cm	0.476	0.238–0.954	0.036
RA-ESA, cm^2^	0.993	0.931–1.059	0.83

HR: hazard ratio; CI: confidence intervals; MACE, major adverse cardiac events. Table footnotes are otherwise identical to Tables 1 and 2

Several multivariate Cox regression models were conducted to determine the independent predictors of MACE including parameters with *p* < 0.05 in the univariate analysis. Baseline model includes NYHA (≥ II), NT-proBNP (≥ 681 pg/mL), MAPSE and LV-EF (model 1). Multivariate Cox regression analysis identified GCW and PMW as independent predictors of MACE after adjusting for the significant parameters listed in Table 4. In contrast, LV-EF and GLS did not remain independent predictors when GCM or PMW were included in the model. Adding GLS to models with significant variables did not improve the model’s predictive power (χ² difference = 1.9, *p* > 0.05). However, incorporating GCW (χ² difference = 8.3, *p* < 0.01) or PMW (χ² difference = 5.6, *p* < 0.05) significantly enhanced the model’s predictive capability.

**Table 4:** Multivariate Cox regression models

Parameter	HR	95% CI	*P*
**Model 1: Baseline**(χ^2^ = 25.3)			
NYHA class, ≥II	1.709	0.744–3.928	0.21
NT-proBNP > 681, pg/mL	1.757	0.735–4.198	0.21
MAPSE	0.818	0.218–3.062	0.77
LV-EF, %	0.948	0.907–0.991	0.019
**Model 2: Baseline + GLS (χ**^**2**^ **= 27.2**, *p* > 0.05)			
NYHA class, ≥II	1.607	0.692–3.730	0.27
NT-proBNP > 681, pg/mL	1.814	0.749–4.394	0.19
MAPSE	1.322	0.299–5.848	0.71
LV-EF, %	0.968	0.915–1.023	0.25
GLS, -%	0.914	0.791–1.055	0.22
**Model 3: Baseline + GLS + GCW < 1**,**238 mm Hg% (χ**^**2**^ **= 35.5**, *p* < 0.01)			
NYHA class, ≥II	1.899	0.829–4.353	0.13
NT-proBNP > 681, pg/mL	1.706	0.731–3.984	0.22
MAPSE	1.828	0.406–8.234	0.43
LV-EF, %	0.973	0.918–1.031	0.36
GLS, -%	1.011	0.865–1.181	0.89
GCW < 1,238 mm Hg%	4.800	1.569–14.681	0.006
**Model 4: Baseline + GLS + PMW < 1**,**286 mm Hg% (χ**^**2**^ **= 32.8**, *p* < 0.05)			
NYHA class, ≥II	1.739	0.757–3.997	0.19
NT-proBNP > 681, pg/mL	1.514	0.639–3.583	0.35
MAPSE	1.592	0.348–7.286	0.55
LV-EF, %	0.969	0.915–1.026	0.28
GLS, -%	1.000	0.852–1.174	0.99
PMW < 1,286 mm Hg%	3.706	1.181–11.629	0.025

HR: hazard ratio; CI: confidence intervals; MACE, major adverse cardiac events. Table footnotes are otherwise identical to Tables 1 and 2

**Fig. 4:**
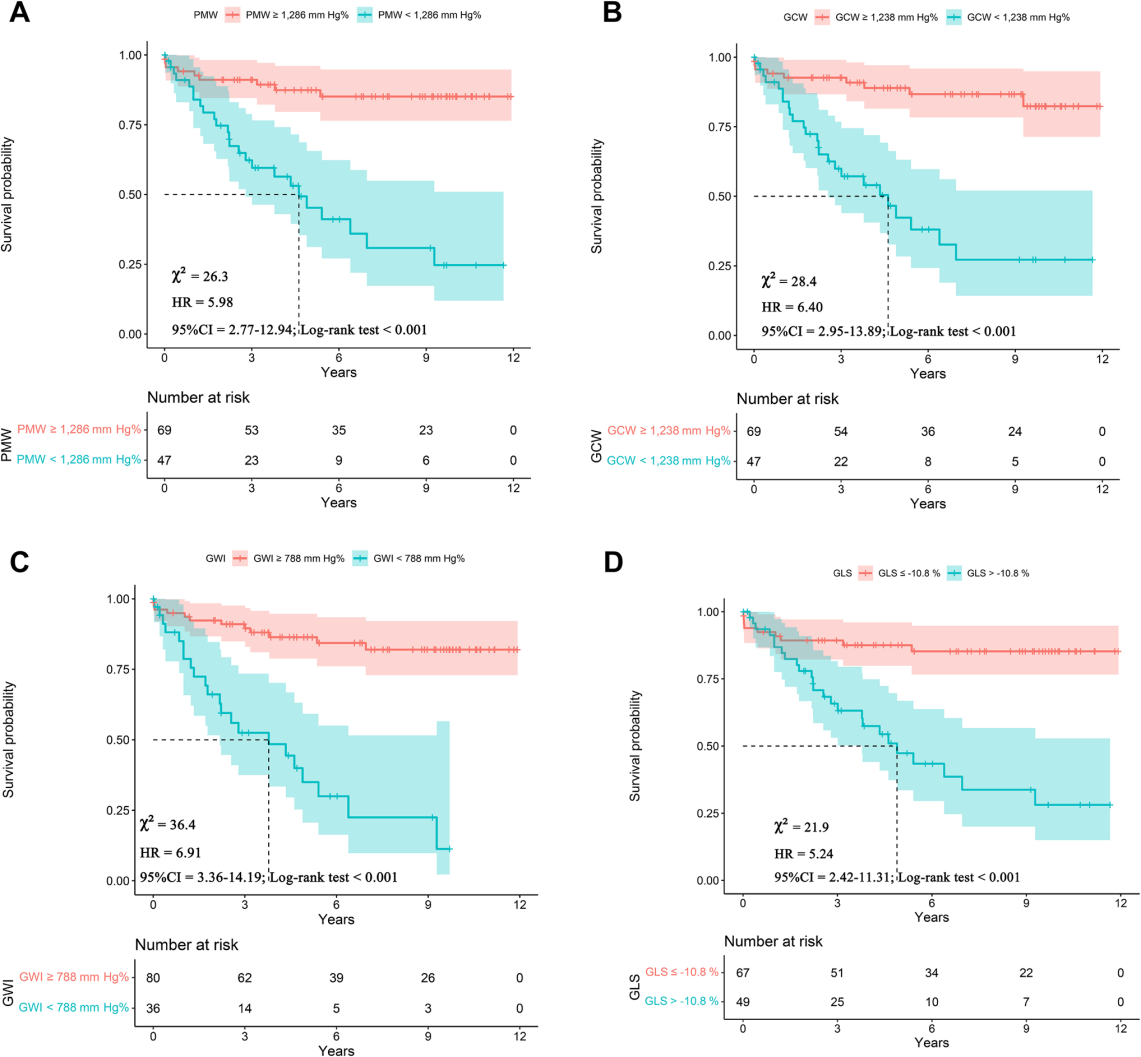
Kaplan-Meier survival curves displaying the occurrence of MACE over time for PMW (panel A), CGW (panel B), GWI (panel C) and GLS (panel D). MACE: major adverse cardiac events, PMW: peak myocardial work index, GCW: global constructive work, GWI: global work index, GLS: global LV longitudinal strain

Results of the Kaplan-Meier analyses are provided on Fig. 4

.

## Discussion

To our knowledge, this is the first study to introduce a simplified nonproprietary formula to assess myocardial work using systolic cuff blood pressure and GLS. We show here that PMW had an excellent correlation with GCW in DCM patients and healthy controls. On one hand, the diagnostic capacity of PMW in differentiating DMC patients from healthy controls are comparable with GCW, outperforming other parameters like GLS and MAPSE, on the other hand, PMW was an independent factor for predicting adverse outcomes in DCM patients.

Invasively measured pressure-volume loops are the gold standard for assessing myocardial performance [20]. Cardiac imaging methods are essential for the evaluation of LV function and assessing the cardiovascular response to treatment. Since direct measurement of LV pressure is only possible invasively, echocardiography depends on surrogate parameters such as LV-EF and LV-GLS [21]. LV-EF as a marker of LV function is influenced by geometry, is load-dependent and shows a high intra- and interobserver variability [21, 22]. Best agreement of volumes measured by echocardiography and cardiac CMR are achieved by 3D echocardiography [12]. Myocardial tissue Doppler strain rate by has a linear relationship with invasively measured LV dp/dt and is among the most feasible clinical markers of contractility [23]. LV dp/dt may be assessed noninvasively, but this is only possible in the presence of mitral regurgitation, where it may be estimated from the rate of velocity rise of the regurgitant jet [24].

Myocardial 2D and 3D strain imaging provides complementary information to LV-EF, as it allows the quantification of segmental as well as global function (LV-GLS). Comparing different methods for the assessment of longitudinal, radial and circumferential strain, best agreement between speckle-tracking strain by echocardiography and feature-tracking by cardiac CMR was observed for GLS [25, 26]. Like LV-EF, GLS is load-dependent. Noninvasive myocardial work index was introduced recently as a modality to overcome afterload dependency, combining LV strain with an estimate of LV pressure obtained by brachial cuff SBP in the absence of outflow/valvular obstruction, allowing the building of LV pressure-strain loops [17, 26]. Its diagnostic and prognostic value has been validated in several hemodynamic conditions. We found that GWI and GCW were not only independent factors for predicting MACE in DCM, but also were the strongest parameters associated with worse long-term outcomes. Furthermore, myocardial work indices provided incremental values to LV-EF and LV-GLS for predicting MACE [15]. Similarly, previous studies have demonstrated that GCW and GWI are independent predictors of MACE in patients with heart failure and reduced ejection fraction (HFrEF), offering additional prognostic value beyond conventional metrics such as GLS and EF [14, 27]. GCW, in particular, has been shown to correlate strongly with myocardial fibrosis and provides superior accuracy in predicting adverse outcomes in DCM patients, supporting its use as a valuable tool for risk stratification [28]. In our study, PMW showed stronger correlation with GCW than with GWI, which is reasonable given that GCW and PMW both represent constructive myocardial work, while GWI reflects total work as the area under the PSL.

The PMW index presented here has several potential clinical implications for clinical use. It provides a quite simple method for assessing myocardial systolic performance, allowing subtle detection of cardiac dysfunction in situations where vendor-specific high-end echocardiography systems are not available. Additionally, PMW outperforms GLS and MAPSE in differentiating patients with DCM from healthy controls, which could be used to monitor disease progression in patients with subclinical conditions and provides an effective means of detecting changes in myocardial function over time. Given its strong correlation with myocardial work indices, PMW may serve as a potential further surrogate for the assessment of systolic function for clinical trials, with the option of easy translation into clinical routine.

### Limitations

This was a single-center, retrospective study with limited sample size. We have only validated the comparable ability of PMW and GCW to differentiate between DCM patients and healthy controls. Large prospective cohort studies are needed to validate the role of PMW in hypertension, heart failure with preserved ejection fraction and other different hemodynamic conditions, such as hypertrophic nonobstructive cardiomyopathy. Another limitation of our study is that we did not assess GWE, which reflects both constructive and wasted work. Since GWE is a known prognostic marker of MACE, this should be considered when interpreting the value of PMW. Additionally, some patients were excluded due to suboptimal acoustic window, which may have introduced selection bias.

## Conclusion

Our present study introduces a method by strain echocardiography using a simplified formula to assess myocardial systolic work. We demonstrated that the diagnostic capacity of PMW index to differentiate between DMC patients and healthy controls are comparable with GCW and outperform other parameters like LV-GLS and MAPSE. PMW may serve as a potential surrogate for predicting adverse outcomes in myocardial diseases.

## Data Availability

No datasets were generated or analysed during the current study.

## Abbreviations

DCM: Non-ischemic dilated cardiomyopathy
DBP: Diastolic blood pressure
GCW: Global constructive work
GLS: Global longitudinal strain
GWE: Global work efficiency
GWI: Global work index
GWW: Global wasted work
LV-EF: Left ventricular ejection fraction
LV-PSL: Left ventricular pressure-strain loop
MACE: Major adverse cardiovascular events
MW: Myocardial work
NYHA: New York Heart Association functional classification
SBP: Systolic blood pressure
STE: Speckle-tracking echocardiography
